# Placenta Percreta Complications

**DOI:** 10.7759/cureus.18842

**Published:** 2021-10-17

**Authors:** Danyon J Anderson, Hefei Liu, Devesh Kumar, Mit Patel, Simon Kim

**Affiliations:** 1 Medicine, School of Medicine, Medical College of Wisconsin, Wauwatosa, USA; 2 Urology, University of Colorado, Aurora, USA

**Keywords:** urinary bladder perforation, disseminated intravascular coagulation (dic), surgical management of obstetrical hemorrhage, placenta accreta syndrome, placenta percreta

## Abstract

Placenta percreta is the most severe form of placenta accreta and is characterized by placental invasion through the entirety of the myometrium and possibly into extrauterine tissues. It is associated with prior cesarean deliveries and placenta previa. Herein, we present the case of a patient who developed placenta percreta and experienced massive blood loss of 27 liters. She developed many complications over the next 11 months, including deep vein thrombosis, pulmonary embolism, preeclampsia after pregnancy, hematoma, blood clots in the bladder, lactation failure, ileus, vesicovaginal fistula, excessive scar tissue requiring surgery, loss of an ovary, and recurrent bladder perforation. We analyze the mechanisms of these complications and the most common complications associated with placenta percreta.

## Introduction

Placenta accreta is a spectrum of potentially fatal adherent placental disorders in pregnant women [1]. Placenta percreta is a specific subtype of placenta accreta. Roughly 5% of placenta accreta cases are percreta. Placenta percreta is associated with higher maternal morbidity than the other placenta accreta subtypes (e.g., placenta accreta vera and placenta increta) due to more extensive invasion [2]. Compared to the two other subtypes, placenta percreta exhibits villous invasion through the entire myometrium and possibly into surrounding structures, such as the bladder [1]. In addition to local destruction, this condition can cause massive hemorrhage, disseminated intervascular coagulopathy, adult respiratory distress syndrome, and kidney failure [3-4]. The cause of placenta percreta is idiopathic but associated with a previous history of cesarean section, placenta previa, grand multiparity, uterine curettage, and Asherman syndrome [2]. Currently, cesarean hysterectomy immediately after childbirth is the gold standard treatment [5]. More conservative management includes leaving the placenta adherent to the uterus. This avoids hysterectomy but requires long-term monitoring for risk of bleeding and thrombotic complications, as well as infections [1-2]. Finally, one-step conservative surgery is an option that involves resecting the invaded area, including the bladder, before resecting the placenta and reconstructing the myometrium [6]. Overall, management of placenta percreta is challenging and may result in lethal complications. Herein, we describe a case of placenta percreta with invasion into the bladder wall and bilateral ureters. The patient suffered multiple systemic complications post-hysterectomy, resulting in large amounts of blood loss and urologic dysfunction. 

## Case presentation

**Figure 1:**
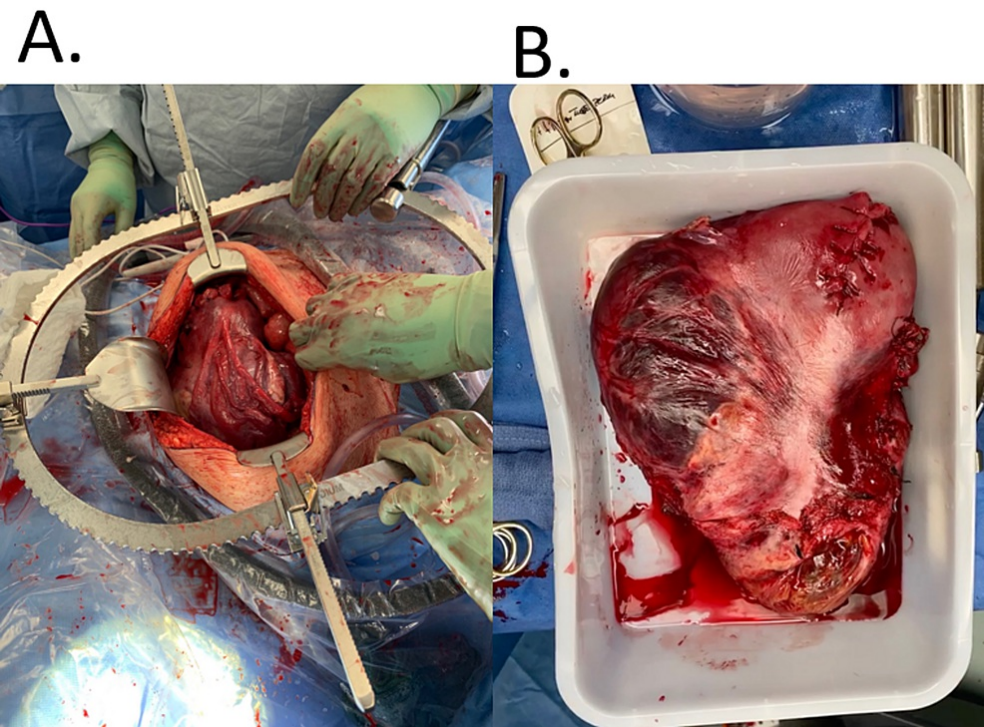
Placenta Before (A) and After (B) Placentectomy A) View of placenta after initial incision during placentectomy; B) Picture of excised placenta taken in operating room

A 38-year-old woman (gravida 4, para 3), with a history of Hashimoto’s thyroiditis, preterm delivery, and cesarean section was referred to the University of Colorado Hospital for management of placenta percreta. Her placenta percreta was diagnosed via ultrasound during a routine examination at 20 weeks gestation. The patient was followed closely by her obstetric team, and no complications arose prior to delivery, except placental invasion. At 34 weeks gestation, the patient was put under general anesthesia in preparation for a cesarean section, hysterectomy, and placentectomy. The cesarean section was uncomplicated and a healthy neonate was delivered weighing 2,807 gm. Visualization of the placenta revealed invasion into the bladder and bilateral ureters (Figure 1). Removal of her uterus, placenta, and cervix was complicated by 27 liters (L) of blood loss, necessitating the transfusion of 51 units of blood; some blood was O+ despite the patient being O-. Her ureters and bladder were partially resected while removing the placenta. After placental removal, her ureters were reimplanted to her bladder. The surgery lasted seven hours and involved six anesthesiologists and seven gynecologic oncologists. 

Post-surgery, the patient remained on a ventilator for 24 hours. She stayed in the hospital for two weeks. During this time, numerous bladder blood clots clogged her catheter, necessitating continuous bladder flushing. A cystoscopy clot evacuation fulguration was performed, and her bladder was cauterized. The patient developed postoperative ileus requiring nasogastric tube placement and making the patient nothing per oral (NPO). A 10" x 2" x 2" periumbilical hematoma developed so she underwent intravascular embolization and was transfused with four units of blood. When bleeding was detected, sterile compressed sponges were applied for hemostasis. The patient developed a pulmonary embolism, which was left untreated due to bleeding risk. She developed lactation failure. She also developed postpartum preeclampsia.

The patient was discharged home with a catheter. Four weeks later, she developed a vesicovaginal fistula. Months later, the patient underwent surgery to get rid of scar tissue. One of her ovaries was removed because it could not be separated from scar tissue. During this surgery, an omental flap was used to repair the vesicovaginal fistula. A Boari bladder flap was used to attach the ureter to the bladder. The patient had perforations in her bladder for 11 months after giving birth.

## Discussion

We present a case of placenta percreta that required 51 units of blood to be transfused, a 30-day hospitalization, multiple surgeries to treat complications, and six anesthesiologists and seven surgeons during the initial procedure. This case illustrates the importance of prenatal diagnosis of placental percreta and preparation for surgery at an adequately resourced hospital. This patient survived because of being prenatally diagnosed and receiving her hysterectomy at a major academic hospital. This case also shows a complicated, extended course post-surgery, revealing the magnitude and severity of possible placenta percreta complications. 

This case provides a framework for analyzing mechanisms of placenta percreta complications (Table 1). This patient’s complications included heavy bleeding, deep vein thrombosis, pulmonary embolism, preeclampsia after pregnancy, hematoma, blood clots in the bladder, lactation failure, ileus, vesicovaginal fistula, excess scar tissue requiring surgery, loss of an ovary, and recurrent bladder perforation which took 11 months to resolve. The most common complication of placenta percreta is heavy bleeding [7]. Our patient lost 27 L of blood. This blood loss occurred due to the placenta being a highly vascular organ. Thus, a retained placenta continues to spill blood until it is removed. In addition, in the placenta percreta, the placenta invades through the entire endometrium and possibly into extrauterine tissue. As it invades, it can invade into blood vessels and cause even more blood loss upon surgical dissection. The second most common major complication of placenta percreta is disseminated intravascular coagulopathy (DIC). In DIC, clotting factors are rapidly consumed causing both clotting and bleeding symptoms. Although our patient was not diagnosed with DIC, she exhibited both clotting symptoms (deep vein thrombosis, pulmonary embolism) and bleeding symptoms (periumbilical hematoma). In the placenta percreta, DIC is often caused by the release of tissue thromboplastin from the retained placenta [8]. The patient’s blood clots in the bladder occurred due to bleeding in the bladder from placental invasion. The patient’s lactation failure occurred due to bleeding out of prolactin and other hormones. Losing 27 L of blood results in the vast majority of your blood coming from blood transfusions and intravenous (IV) fluids which are devoid of hormones present in the blood that was lost. Ileus, as seen in our patient, is a well-reported surgical complication. Fistulas can occur following trauma and inflammation and are documented complications of both placenta accrete and hysterectomy [9]. Ovarian loss was caused by ovarian fibrosis and removal of fibrosed tissue. Recurrent bladder perforation occurred due to repeated microtrauma from bladder staples that had been placed to control the massive bleeding. As the bladder expanded and contracted, it changed shape, leading to the staples putting dynamic pressure on the bladder and perforating it at times. We hope that our patient’s case will help to outline the complications of placenta percreta and elucidate their mechanisms.

**Table 1:** Mechanisms of Placenta Percreta Complications

Complication	Mechanism(s)
Heavy Bleeding	Retained placenta (which is highly vascular). Placental invasion into the extraplacental vasculature.
Disseminated Intravascular Coagulopathy (DIC)	Release of tissue thromboplastin from retained placenta.
Hematuria	Invasion of placenta into the bladder, causing bleeding.
Lactation Failure	Massive loss of blood causes loss of hormones that are carried in the blood, including prolactin.
Fistulas	Trauma and inflammation can cause fistulas. Trauma in the region of the bladder can cause a vesicovaginal fistula.
Ovarian Loss	Scar tissue from the hysterectomy covered the ovary and needed to be removed.
Ileus	Well-documented surgical complication.
Bladder Perforation	Bladder staples (needed to control rapid, massive bleeding) caused repeated microtrauma to the bladder as the bladder expanded and contracted.

## Conclusions

Our patient had many complications arise from her placental percreta. For this reason, her case offers a thorough illustration of complications of placenta percreta. Common complications of placenta percreta include heavy bleeding, DIC, blood clots in the bladder, lactation failure, fistulas, ovarian loss, ileus, and recurrent bladder perforations. The mechanisms of these complications are described in the text.
